# Deep weathering in the semi-arid Coastal Cordillera, Chile

**DOI:** 10.1038/s41598-021-90267-7

**Published:** 2021-06-22

**Authors:** Laura V. Krone, Ferdinand J. Hampl, Christopher Schwerdhelm, Casey Bryce, Lars Ganzert, Axel Kitte, Kirstin Übernickel, Armin Dielforder, Santiago Aldaz, Rómulo Oses-Pedraza, Jeffrey Paulo H. Perez, Pablo Sanchez-Alfaro, Dirk Wagner, Ute Weckmann, Friedhelm von Blanckenburg

**Affiliations:** 1 GFZ German Research Centre for Geosciences, Potsdam, Germany; 2 Department of Applied Geochemistry, Technische Universität Berlin, Berlin, Germany; 3 Department of Geosciences, Eberhard Karls Universität Tübingen, Tübingen, Germany; 4 Department of Earth Sciences, University of Bristol, Bristol, UK; 5 CRIDESAT, Universidad de Atacama, Copiapó, Chile; 6 Instituto de Ciencias de La Tierra, Universidad Austral de Chile, Valdivia, Chile; 7 Institute of Geosciences, Universität Potsdam, Potsdam, Germany; 8 Institute of Geological Sciences, Freie Universität Berlin, Berlin, Germany; 9 Present Address: Institute of Geology, Leibniz Universität Hannover, Hannover, Germany

**Keywords:** Environmental sciences, Geochemistry

## Abstract

The weathering front is the boundary beneath Earth’s surface where pristine rock is converted into weathered rock. It is the base of the “critical zone”, in which the lithosphere, biosphere, and atmosphere interact. Typically, this front is located no more than 20 m deep in granitoid rock in humid climate zones. Its depth and the degree of rock weathering are commonly linked to oxygen transport and fluid flow. By drilling into fractured igneous rock in the semi-arid climate zone of the Coastal Cordillera in Chile we found multiple weathering fronts of which the deepest is 76 m beneath the surface. Rock is weathered to varying degrees, contains core stones, and strongly altered zones featuring intensive iron oxidation and high porosity. Geophysical borehole measurements and chemical weathering indicators reveal more intense weathering where fracturing is extensive, and porosity is higher than in bedrock. Only the top 10 m feature a continuous weathering gradient towards the surface. We suggest that tectonic preconditioning by fracturing provided transport pathways for oxygen to greater depths, inducing porosity by oxidation. Porosity was preserved throughout the weathering process, as secondary minerals were barely formed due to the low fluid flow.

## Introduction

Rock weathering, the conversion of coherent rock through contact with atmospheric gases, water, or organisms into weathered rock and mobile soil is a fundamental geologic process. Weathering disintegrates rock for transport by erosion^1^ and consumes global atmospheric CO_2_, thereby modulating global climate over geological timescales^2^. In addition, the release of nutrients by weathering provides nutrients for microorganisms and plants^3^. The deep weathering zone is the lower part of the “critical zone”, which is defined as the section of Earth’s surface extending vertically from the depth where weathering begins to the top of the vegetation canopy, the zone “where rock meets life”^4^.

The thickness of weathering zones occurs in a defined depth interval for given lithologies in eroding landscapes^5^. In felsic lithologies, the weathering zone and associated reaction fronts are much thicker and found deeper than in mafic lithologies^5,6^. This observation requires the advance of the weathering front at depth to be coupled to erosion at the surface through a feedback^5,7^. Recent studies suggest that the locus of the weathering front is governed by the maximum depth of O_2_ diffusion^8^ or the depth of the saturated zone^9,10^ besides the lithological precondition^6^. Deeper still, groundwater may induce localised weathering up to 250 m and consequentially may have significant impact on weathering^11^.

Yet, pathways are needed that connect the depth with the surface to transport fluid and gaseous reactants to depth. Fractures and porosity serve as such pathways for diffusive and advective transport^6,8,12^. Weathering-induced fracturing enhances the porosity and acts as a positive feedback in propagating the weathering front to depth^8,13,14^. In particular, it drives Fe oxidation generating strain that leads to the formation of fractures^6,14–16^. Due to advancing weathering, secondary mineral formation may in turn fill pores^13^, a process that inhibits a deeper advance of the weathering front^12^. Also, the activity of chemolithoautotrophic microorganisms contributes to weathering at depth^17^ as well as other microorganisms such as fungi^18^.

Non-weathering-related processes generate pathways resulting from tectonic pre-fracturing^19,20^, which often involves the development of planar faults and macrofractures on the metre-scale^21^. Such macroscale structures are thus distinct from weathering-induced fractures that are typically developed on nano- to micro-scale within mineral grains and along grain boundaries^22^. Nevertheless, tectonic pre-fracturing also causes microfractures in minerals^23^.

Weathering fronts in granitoid rocks exposed to humid climate are typically observed at about 10–20 m depth^5,6,12,24–26^. Studies on the depth of the weathering front in semi-arid and arid climate though are rare. Vázquez et al.^27^ and Stierman and Healy^28^ reported much deeper weathering fronts of 30 and 70 m for Central Chile and the Mojave Desert, USA, respectively. These sites both have a similar climate and are located close to an active plate boundary and have thus experienced tectonic deformation. Even though tectonic processes are thought to promote later weathering through fracturing^19^ whether they also result in deeper weathering is still an open question. In this regard, locations in dry climate are particularly promising to disclose processes that promote weathering and that set the depth of the weathering front, as their imprints are impacted by only minimal amounts of fluid.

To close this knowledge gap, this study aims to identify the depth and degree of weathering in granitoid rock in a semi-arid climate. Here, we present the first results of a drilling campaign conducted in the framework of the “EarthShape” project at the field site Santa Gracia (Coastal Cordillera, Chile)^29^. We drilled an 87 m deep well and performed geological, geochemical, and geophysical investigations. In further studies the samples will be used to investigate the diversity and impact of microbial communities for deep weathering fronts, and thus full contamination control was employed during drilling. In this paper, we present a description of drilling activities, results on the porosity, specific surface area, the geometry of fractures, Fe oxidation, the degree of chemical weathering, volumetric strain, and the weathering rate using cosmogenic nuclides. We provide a first interpretation of the impact of fracturing based on these weathering features and borehole geophysics.

## Geological setting and drilling procedure

### Geological setting

**Figure 1:**
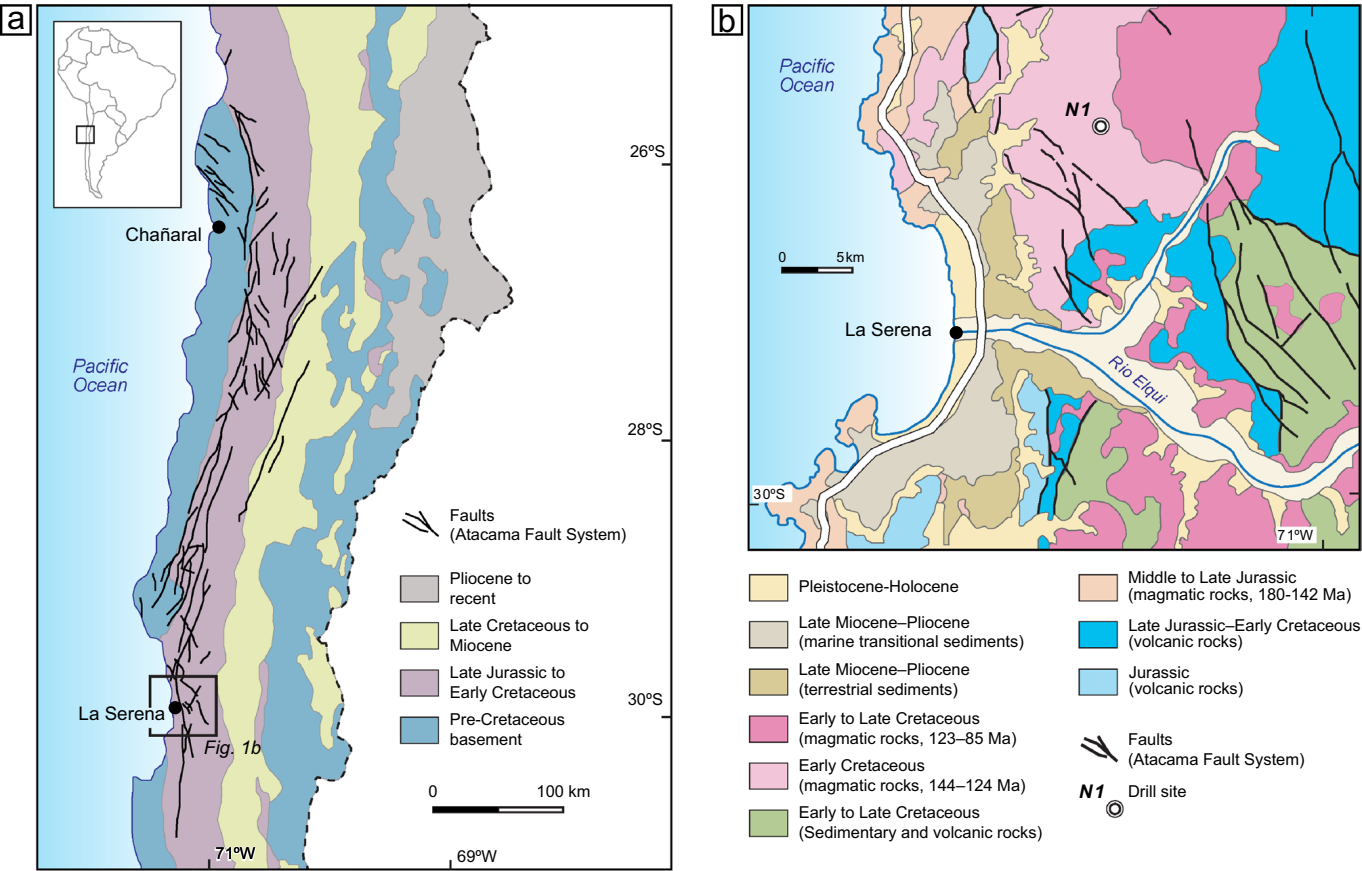
(**a**) Simplified geological map of northern Chile between 25 °S and 31 °S indicating the Atacama Fault System in the present-day forearc as based on maps in Tornos et al.^61^. Traces of the Atacama fault system are based on Cembrano et al.^31^. The study area near La Serena is indicated by the black rectangle. (**b**) Simplified geological map of the study area near La Serena as based on SERNAGEOMIN^30^. The NW- and N-trending faults are features of the southernmost part of the Atacama Fault System.

The Chilean Coastal Cordillera is the westernmost mountain range of the Andean orogen and extends from north to south along the Pacific coast of South America. In northern Chile, the elevation of the range is about 0.5–2 km and its width up to 50 km. The lithological basement units exposed in the Coastal Cordillera were part of the Jurassic to early Cretaceous magmatic arc, comprising plutons of granitoid composition and andesitic volcanic rocks (Fig. 1a)^30^. At present, the Coastal Cordillera is situated within the forearc. One of the largest tectonic structures of the Coastal Cordillera is the Atacama Fault System (AFS) that can be traced for more than 1000 km between Iquique at 20°S and La Serena at 30°S^31,32^ (Fig. 1a). This fault system consists of steeply dipping, northwest, north, and northeast oriented strike-slip faults which are striking over tens to hundreds of kilometres. The faults formed mainly during the Late Jurassic and Early Cretaceous and record predominantly sinistral arc-parallel strike-slip movements suggesting that the AFS accommodated oblique plate convergence^31,33^. Initial deformation was ductile under amphibolite to greenschist facies conditions, followed by brittle deformation during the mid-Cretaceous. Cooling and exhumation of the magmatic arc occurred mainly during the late Mesozoic development of the AFS, but also during the Cenozoic phase of Andean mountain building^31,32,34^. Faulting in the study area along N-S trending faults continued at least until the Early Miocene and resulted in an uplift of the eastern Cordillera relative to the western part^35^. This study’s drill site northeast of La Serena is located in the western Coastal Cordillera in an Early Cretaceous pluton of dioritic to granodioritic composition (Fig. 1b). The pluton is faulted by steeply dipping, northwest to northeast oriented faults that can be traced over several kilometres and represent the southernmost part of the AFS.

The Early Cretaceous activity of the AFS was coupled to the formation of the “Chilean Iron Belt”, a cluster of mineral deposits of Cretaceous age that is spatially and genetically related to the AFS. This cluster is rich in iron oxide, copper, gold, iron oxide, apatite, and stratabound silver deposits, which are hosted in thick mafic-to-intermediate subaerial volcanic units of Middle Jurassic to Early Cretaceous age^36^. Hydrothermal alteration in the country rocks is extensive and consists of a mineral assemblage of actinolite, scapolite, biotite, tourmaline, chlorite, chlorapatite, sphene, minor amounts of garnet, and pyrite. One of the largest iron oxide–apatite deposits of the coastal range, “El Romeral”, is located 10 km to the northwest of the study site. The deposit formed about 128 Ma during extensional sinistral NNW strike-slip faulting along the AFS^36^. There is no documentation on supergene formation processes in the study area, however such processes have been documented for the Atacama Desert further north^36,37^. There, supergene processes have been dated using alunite-group minerals indicating an extended period between ~ 45 and 9 Ma of supergene oxidation, with a peak at ~ 21–14 Ma that is dominated by downward circulation of meteoric water under semi-arid to arid climate conditions^37^.

The study site Santa Gracia (29.76° S, 71.16° W) is characterised by semi-arid climate with a mean annual precipitation of 87 mm a^−1^ and mean annual temperature of 16.1 °C^38^. The sparse vegetation is dominated by shrubs and cacti and is highly influenced by livestock grazing. Luebert and Pliscoff^39^ describe the native vegetation in Santa Gracia as Mediterranean desertic shrubs. Dominant plant species are *Proustia cuneifolia*, *Senna cumingii* and *Cordia decandra* for shrubs and *Cumulopuntia sphaerica* and *Eulychnia acida* for cacti^29^. Soils in the study area are thin with an A and B-horizon thickness of 30–55 cm, underlain by saprolite^29^. The drill sites are located on a ridge at 622 m. a. s. l., surrounded by hillslopes with dipping angles of 5°–20°.

### Drilling procedure

The wireline diamond drilling of hole N1 was conducted in March and April 2019, using a standard industry truck-mounted PQ3-sized (85 mm core diameter, 123 mm hole diameter) rotary drilling rig (Sondajes Araos E.I.R.L.). A Long Year Series 4 diamond drilling crown suitable for abrasive rock and a Long Year Series 9 crown for moderately abrasive rock were used. The final true vertical well depth reached 87.2 m, using a standard wireline continuous coring system recovering up to 1.5 m long core runs contained in stainless steel liners. Potable water with added contamination control tracer (fluorescent particles at a size range similar to microorganisms, according to the procedure of Friese et al.^40^) was used to drill and to monitor potential contamination of samples by microorganisms introduced by the drilling fluid. The contamination control is necessary for microbiological studies to be conducted on these samples. A detailed description of the contamination control and the results can be found in the methods section and the results in the data supplement (Table S8^41^).

Increasing sample recovery in the uppermost unconsolidated saprolite was attempted by using additives (AMC GEL XTRA bentonite, AMC CR 650 polymer, both Imdex Ltd, Australia) in the topmost 11.45 m was unsuccessful and was omitted for the remainder of the coring to avoid contamination (see “Methods”). The circulating drilling fluid was recycled by decantation into a settling pit. Due to substantial drilling fluid losses into the rock fresh potable water was added daily to the circulating system. To stabilize the borehole in the shallow section, a conductor casing was installed from surface to 6.92 m depth. The average rate of penetration was 3.32 m day^−1^ in soil and saprolite, and up to 6.45 m day^−1^ in rock. The average recovery was 1.67 m day^−1^ in soil, and 6.45 m day^−1^ in crystalline rock. Because of low recovery in the uppermost soil and loose saprolite two additional boreholes N1C (surface—5.6 m) and N2 (surface—6.85 m) were cored by a hammer sampler equipped with a core catcher (“cuchara española”, Spanish spoon) with a maximum run length of 500 mm and a diameter of 30 mm. Both holes N1C and N2 were drilled from 5.6 and 6.85 m, respectively, to a final depth of 10 m with the rotary drilling and wireline coring equipment described above. The drilling advance with the hammer technique was 2.5 m day^−1^ and the average recovery was 1.46 m day^−1^. After completion of the drilling, geophysical well logging data was acquired using downhole wireline logging tools.

Mineralogical, petrophysical, and geochemical analytical procedures are described in the methods section. All data are contained in an accompanying data publication^41^ and tables therein are here referred to as Tables S1–S8.

## Results

### Core description

**Figure 2:**
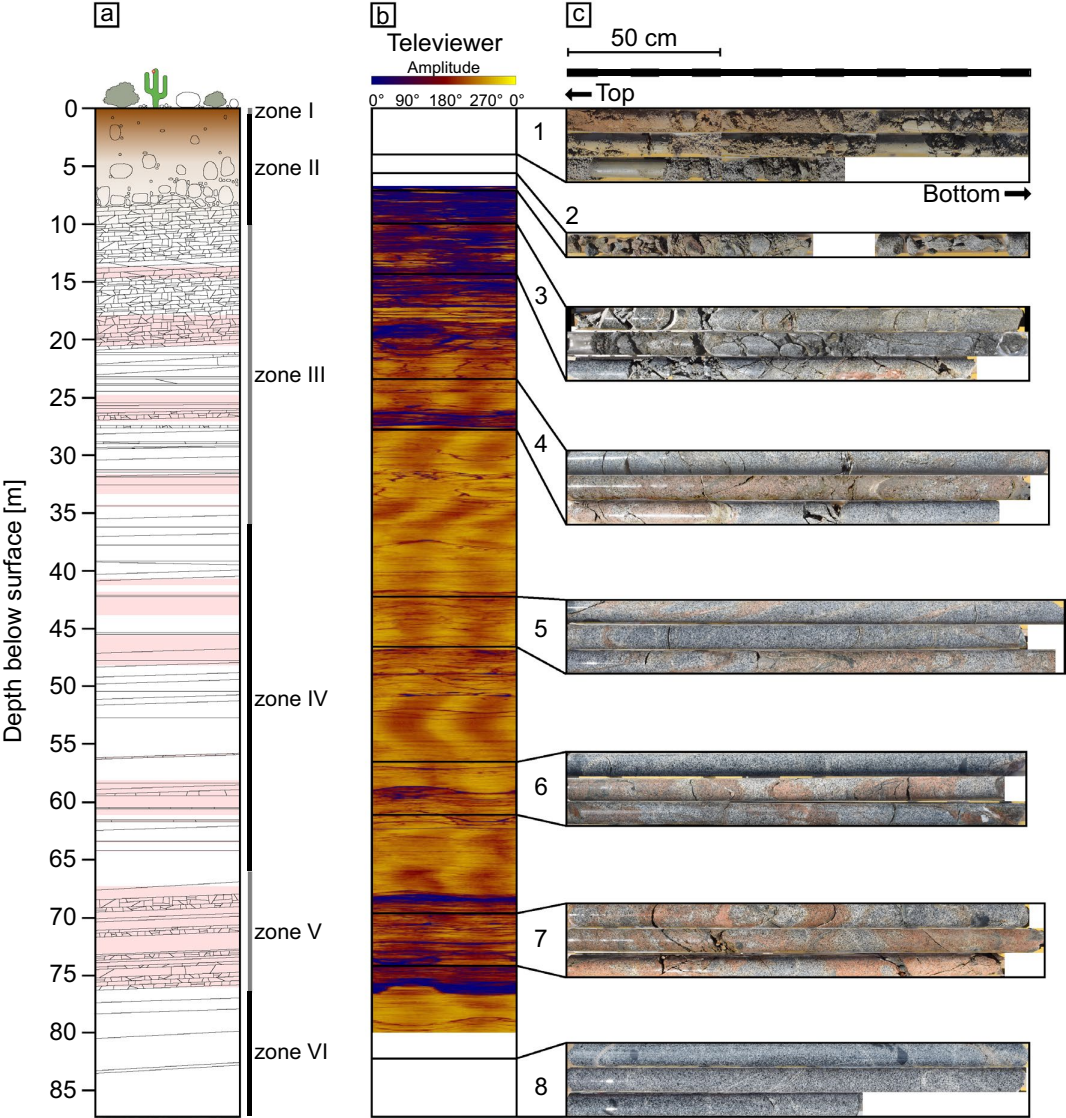
(**a**) Schematic core log compiled from core descriptions and -photos. Brown part in the upper metres indicates soil, pink parts indicate red-stained zones. Black lines indicate fractures but are not showing their orientation. (**b**) Acoustic televiewer image of the unrolled borehole wall from well logging. Fractures are shown as horizontal lines. Oblique fractures were projected onto the 0° azimute angle at their shallowest depth. The colours imply different amplitudes due to the varying velocity of the acoustic signal with blue indicating a low amplitude (0) and yellow a high amplitude (50,000). Details are described in the method section. (**c**) Core photos of selected zones: 1: soil and saprolite (zone I–II); 2: Saprolite (zone II); 3: Transition between saprolite and saprock (zone II–III); 4–7: saprock (zone III–V); 6: fresh bedrock (zone VI). Photos of the uppermost metres of the profile (panel 1) show drill cores from the well N1C.

The drilled core from borehole N1 reveals distinct zones (I-VI) from the surface to bedrock (Fig. 2a). The upper 0.3–0.5 m are soil (zone I), followed by zone II of highly weathered loose saprolite containing core stones to a depth of 10 m. Between 10 and 36 m, we found more consolidated saprolite featuring fractures, red-stained parts, and a moderate porosity (2.8 ± 2%; average ± 1 standard deviation (SD)) in zone III. In zone IV (36 to 66 m), less strongly fractured and only moderately altered rock (or “saprock”) is intermingled with slightly red-stained rock of lower porosity (1.3 ± 1%). In zone V between 66 and 76.5 m, a highly altered, intensely fractured, red-coloured rock featuring high porosity up to 7.5% (average 3.5 ± 3%) was found. Some sections in this zone are of unconsolidated fabric and yield zones of dark red, presumably hydrothermal alteration (Fig. 2c). This zone is rich in what likely are Fe oxides and oxyhydroxides. Zones III to V are referred to as saprock. Zone VI from 76.5 to 87.5 m comprises what appears to be unweathered grey bedrock with low porosity (0.3 ± 0.2%) and only few fractures and zones of red alteration.

**Figure 3:**
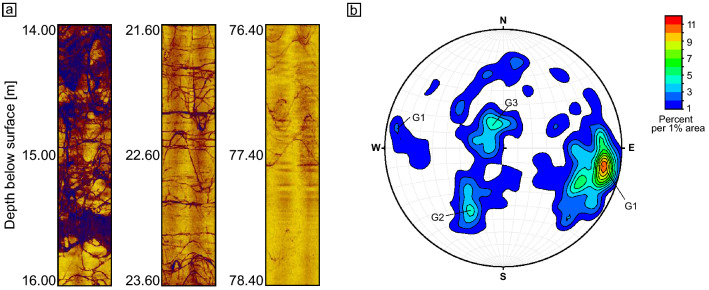
(**a**) Detailed acoustic televiewer images showing different types of fractures, where sinusoidal and planar patterns are mainly attributed to a tectonic origin. Left: Both planar and non-planar intensely branched fractures are forming a network where the rock is subjected to a high degree of weathering. Centre: Planar and non-planar fractures in saprolite of a low weathering degree. Oblique and horizontal planar fractures are intersected. Right: Bedrock featuring neither strong branched nor planar fractures. (**b**) Lower hemisphere stereographic projection illustrating the orientation of fractures as obtained from the televiewer data (poles to fracture planes, *n* = 283). The fractures can be grouped into three groups, G1, G2, and G3. Group 1 is the dominant group and comprises fractures that are steeply inclined and dominantly strike NNE-SSW. Groups 2 and 3 comprise gently to moderately inclined fractures that strike NW–SE. Data plotted with OSXStereonet^62^.

Acoustic televiewer images display weathered zones and brittle rock in blue colours (Fig. 2b, 3a). Fractures are also recognizable on these images: sinusoidal structures indicate inclined fractures whereas planar structures show horizontal fractures (Fig. 3a). Detailed results from all measured downhole logging tools are reported in Weckmann et al.^42^.

### Fracture orientation

Drill cores recovered from well N1 show a dense network of planar fractures with an average spacing of less than 0.5 m. Most fractures are less than a few mm thick. Larger fracture zones can reach a thickness of a few decimetres and are commonly bound to shear zones and faults, or other compositional anisotropies in the magmatic host rock. Fracture orientations were compiled from televiewer data from borehole N1 (*n* = 283). The contour diagram for poles of fracture planes indicates three main sets of fractures (Fig. 3b). The first and dominant group comprises fractures that strike predominantly NNE-SSW and dip at high angle toward the WNW, and to a lesser degree toward the ESE (G1 in Fig. 3b). The second group includes fractures that strike approximately NW–SE and dip at moderate angles toward the NE (G2 in Fig. 3b). In comparison, the third group includes fractures that also strike approximately NW–SE, but dip at low angle toward the SW. In addition, the data comprises some fractures that appear randomly oriented and cannot be assigned to one of the three groups above.

### Bedrock composition and hydrothermal alteration

**Figure 4:**
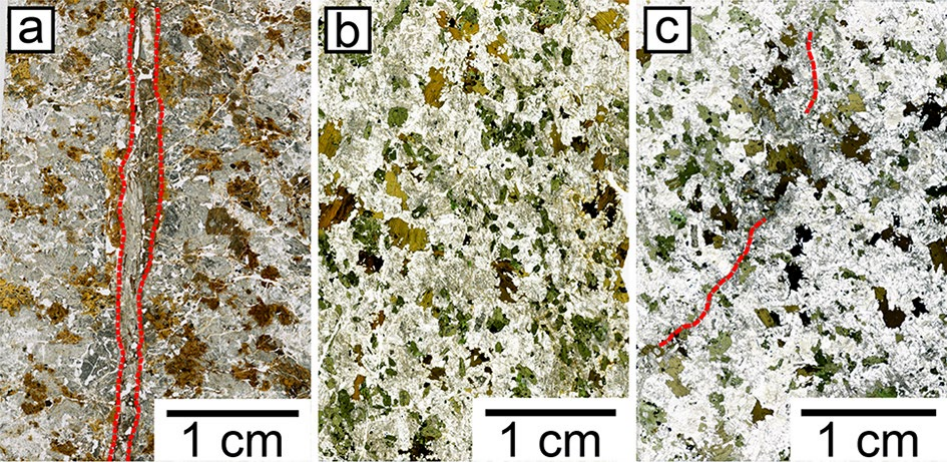
Thin sections of (**a**) The strongest degree of weathering found in a sample from a nearby soil pit showing a carbonate vein delineated in red (sample depth: 1.74 m). (**b**) Moderately weathered sample from zone C (average depth: 11.8 m). (**c**) Bedrock sample (zone VI) with a carbonate veinlet (average depth: 82.6 m).

The primary lithology changes little with depth apart from secondary hydrothermal features (Fig. 4). However, occasional mafic xenoliths and more felsic zones can be found. The bedrock is characterised by ~ 58% SiO_2_, ~ 6% for both Na_2_O and K_2_O, ~ 6% CaO, ~ 3% MgO, ~ 7% Fe_2_O_3_ and ~ 17% Al_2_O_3_, respectively, all in percentages by weight (Table S2). Major and trace elements show slight variations (SD < 13%). Some elements show higher bedrock variations (SD Cu: 27%, Cs: 24%). The average Zr concentration in the bedrock is 166 ± 11 ppm (SD) with a relative uncertainty of 8% calculated from reference material measurements. For comparison, the average Zr concentration from relatively unweathered bedrock specimens collected previously near the study area was 115 ± 68 (SD) ppm^29^. Element distribution maps of a polished bedrock slab were used to calculate a modal mineral composition (in area% as an approximation of volume%) of ~ 44% plagioclase, ~ 16% hornblende, ~ 16% K-feldspar, ~ 11% quartz, and ~ 6% biotite. Accessory minerals are apatite (~ 2%), magnetite (~ 1%), zircon (~ 0.5%), titanite (~ 0.4%), and copper-bearing sulphides (mainly chalcopyrite, ~ 0.3%). Minor abundances of chlorite, calcite, pumpellyite, ilmenite, sericite, and epidote were detected. According to this composition the bedrock can be classified as quartz monzodiorite. Large parts of the core are hydrothermally altered which is evident from the presence of hematite (also martite), chlorite, laumontite, and sericite occurring mainly as a replacement of plagioclase. Anhydrite is present in minor abundance as infilling in veins and fractures and is sometimes intergrown with secondary K-feldspar. Late calcite veins crosscut previous alteration zones. Fracture surfaces in red zones are covered with hematite, chlorite, and carbonates.

### Physical properties

**Figure 5:**
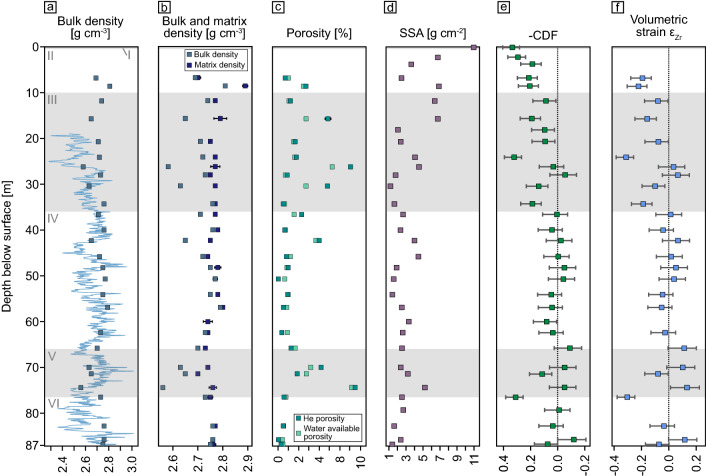
(**a**) Measured bulk density (dark blue) and calculated bulk density from p-wave velocities (light blue). (**b**) Matrix density indicates the rock density without pores; bulk density the density including pore volume. Error bars indicate the standard deviation of 10 replicate measurements of the same sample. (**c**) Porosity measured with He pycnometry (“He Porosity”) and water saturation (“Water available porosity”). (**d**) Specific surface area (SSA) of powdered samples measured by N_2_ sorption analysis using the BET equation. (**e**) Chemical depletion fraction (CDF) shows fractional mass loss relative to the average of four bedrock samples (negative CDF). Dotted reference line for zero mass loss. Values > 0 indicate elemental loss. (**f**) Calculated volumetric strain (ε_Zr_) for the weathering profile. Positive values indicate dilation whereas negative values denote the collapse of the profile. The accuracy of the CDF and of ε_Zr_ is limited by the variability in bedrock Zr concentration which is shown as error bars on all samples in panel (**e**) and (**f**). Grey and white shades denote the core zones (I–VI) described in the results.

The average bulk bedrock density below 80 m is 2.757 ± 0.005 g cm^−3^ (Table S1). In the strongly altered zone V, the average bulk density decreases to 2.64 ± 0.06 g cm^−3^. At 26 m the density is as low as 2.58 g cm^−3^ (Fig. 5a). The uncertainty of density measurements is within 0.2%. We did not capture the decrease in bulk density expected at the surface because our method required coherent samples which also have low porosity. To counter a potential decrease in density the abundance of secondary minerals with higher density also increases towards the surface. This is also reflected in the higher matrix density data (Fig. 5b, Table S1). Densities as low as 2.3 g cm^−3^ were calculated from p-wave velocity measurements. Soil densities from 0 to 60 cm depth are on average at 1.5 g cm^−3^^29^.

The average porosity of bedrock determined by helium (He) porosimetry is 0.3 ± 0.2% (SD). The He porosity is increasing to 7.5% (average 3.5 ± 3%) in the strongly altered zone V. Higher porosity was found at 42 m (4%), 30 m (4.8%), at 26 m (7%), and at 15.5 m (4.8%) (Fig. 5c; Table S1). Surface porosities remain low (0.7–2.7%) as samples needed to be coherent for the measurement and thus do not reflect unconsolidated saprolite. He porosity measurements are within an uncertainty of 1%. Values for water-available porosity are consistent with He porosity, whereas the water-available porosity calculated from saturation is lower than He porosity. Some samples with extremely low He porosity show higher values for water-available porosity; these differences are within uncertainty, however. We assume the water-available porosity reflects the connected porosity.

For bedrock in zone VI, the average value of the specific surface area (SSA) is 2.0 ± 0.5 m^2^ g^−1^ (Fig. 5d). The strongly altered zone V shows higher SSA ranging from 2.36 to 5.21 m^2^ g^−1^. In zones III and IV, higher SSA around 6.5 m^2^ g^−1^ occur locally but most of the measured SSA ranges between 1.5 and 4.5 m^2^ g^−1^. Towards the surface, the SSA increases from 6.0 to 11.0 m^2^ g^−1^ (Table S1).

### Weathering indicators

**Figure 6:**
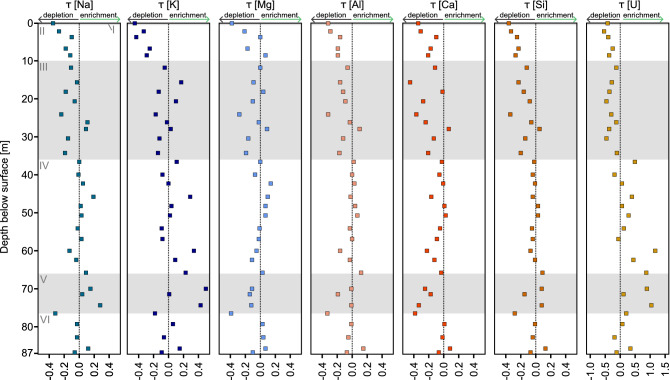
Elemental mass transfer coefficient (τ) calculated relative to four averaged bedrock samples. Values < 0 indicate depletion of an element whereas values > 0 indicate enrichment relative to bedrock. Dotted reference line indicates zero mass loss. Grey and white shades denote the core zones (I–VI) described in the results.

**Figure 7:**
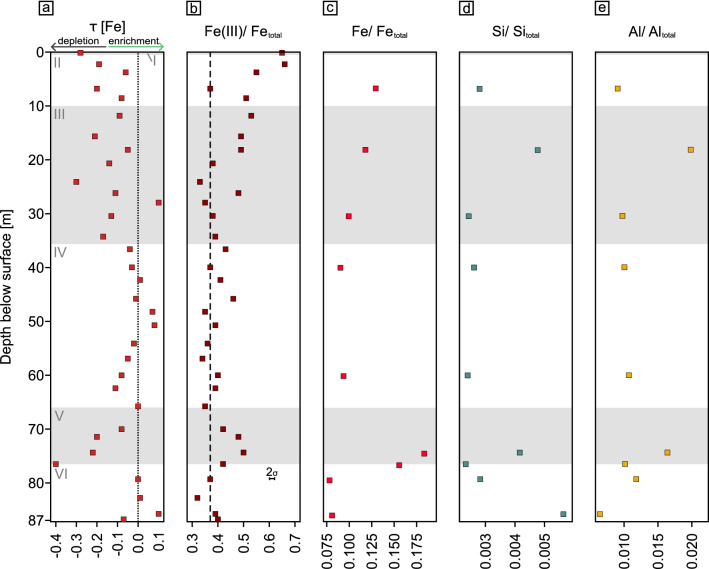
Weathering degree characterization by using Fe, Si, and Al. (**a**) Elemental mass transfer coefficient (τ) of Fe, dotted line indicates zero loss. Values < 0 show relative depletion of Fe, values > 0 enrichment of Fe relative to bedrock. (**b**) Redox state of bulk drill core samples, here shown as Fe(III)/Fe_total_ ratios. Dashed line indicates the average bedrock value of 0.37. (**c**) Ratio of reactive Fe mineral fraction (extractable by citrate bicarbonate dithionite, (CBD)) to total Fe content here expressed as Fe/Fe_total_. (**d**) Si/Si_total_ ratios representing ratio of CBD extractable Si to total Si. (**e**) Al/Al_total_ ratios representing ratio of CBD extractable Al to total Al. Grey and white shades denote the different zones (I–VI) described in the results.

The chemical depletion fraction (CDF, see “Methods”) quantifies the fraction of mass lost by weathering relative to the bedrock. In zone V, CDF values between 0.11 and 0.31 indicate a loss of more soluble elements. Zone IV appears to be virtually unweathered with CDF values between 0.08 and − 0.09. Starting with zone III, the CDF is constantly decreasing towards the surface. At 24 m below the surface (zone III), a high-weathering degree zone with a CDF of 0.32 was found (Fig. 5e; Table S4). A continuous gradient in CDF was only encountered in zone II where also the shallowest sample yielded the lowest CDF of 0.33. Generally, positive CDF values were found where fracturing is more pervasive as seen by televiewer data. The fractional elemental mass loss (τ, see methods) generally reflects the CDF. Most elements are depleted in the deep altered zones III and V, except for potassium (K) and uranium (U). The most strongly depleted elements at the surface compared to the bedrock are K (− 0.46), U (− 0.43), and Ca (− 0.41) (Figs. 6, 7a; Table S4).

Strain (ε_Zr_), the change in volume relative to bedrock^43^, yields values near zero for saprock in zone IV (0.11 to − 0.05). More weathered zones (zone III and V) show variable strain (Fig. 5f). Towards the surface, especially in zone II, the calculated values are negative (− 0.08 to − 0.22), indicating collapse.

### Redox state and extractable oxides

Fe(III)/Fe_total_ in bedrock is 0.37 ± 0.05 (Table S5). An overall trend of increasing Fe(III)/Fe_total_ ratios towards the surface is recognizable, with Fe being more oxidised in almost all weathered samples compared to bedrock (Fig. 7b). In the highly altered zones V and III, Fe(III)/Fe_total_ increases to 0.42–0.50, whereas Fe(III)/Fe_total_ ratios in zone IV depict similar values as bedrock. Highest Fe(III)/Fe_total_ is found at the surface (0.66) where the loss of total Fe is also highest as denoted by τ_Fe_.

In deep bedrock, 3.68 mg Fe g^−1^ was extracted by citrate bicarbonate dithionite (CBD; reducible oxides). In weathered rock the amount of extracted Fe ranges from 4.24 to 8.94 mg Fe g^−1^ rock powder (Fig. 7; Table S5). When compared to the total amount of iron in each sample, we extracted 7.8–18.3% of total iron. With CBD extractable Si concentrations obtained from bedrock are 0.750–1.55 mg Si g^−1^ rock powder. In weathered rock, the concentrations range from 0.552 to 1.25 mg Si g^−1^ rock powder. Extractable Al concentrations in bedrock are 0.516–0.900 mg Al g^−1^ rock powder. In weathered rock they range from 0.750 to 1.51 mg Al g^−1^ rock powder (Fig. 7d,e). Compared to total Si, CBD extractable Si accounts for 0.23–0.56% (CBD). CBD extractable Al accounts for 0.64–1.98% of the total Al.

### In situ ^10^Be

Soil denudation rates from in situ cosmogenic ^10^Be at the drill site range between 24.8 and 35.6 t km^−2^ year^−1^ (mean 29.6 ± 4.0 t km^−2^ year^−1^, *n* = 4). Their integration time is 56 ± 7 kyr (see “Methods”). By comparison, denudation rates from soil pits nearby range from 16 to 22 t km^−2^ year^−1^^44^, whereas catchment-average denudation rates in the study area are 20 to 29 t km^−2^ year^−1^^45^. Using the topsoil CDF of 0.33 a core-integrated weathering rate of 9.9 ± 1.3 t km^−2^ year^−1^ results. Using the CDF of 0.21 at the bottom of zone II (8.6 m depth) a weathering rate integrating from 80 m to 10 of 6.1 ± 0.8 t km^−2^ year^−1^ is obtained.

## Discussion

The main difference between our study site in arid climate and study sites in temperate and humid climate^5^ is the great depth in the granitoid rocks (as deep as 76 m) to which weathering proceeds. Rather than a continuous gradient in weathering indicators, we found several distinct weathering fronts at 20, 36, and 76 m depth that, unlike those set by chemical reactions driven by top-down inputs^6^, repeat each other in style and intensity.

A key observation is that the depletion of the most soluble major elements is concomitant with high fracture density, high porosity, and low bulk regolith density. Moreover, the highest elemental loss is detected in the proximity of planar fractures or fracture zones. In particular, many observed fractures are rimmed by weathered halos. We therefore assume that fractures act as a major pathway for the advective transport of reactants to depth^46^. Many of these fractures are part of the first and second fracture group identified in the televiewer data. The approximate N-S orientation and moderate to high dip angles of these fractures are consistent with the orientation of faults in the study area and the general strike and kinematics of the Atacama fault system. We interpret these fractures to have formed during the Late Mesozoic activity of the fault system and to record damage offside larger faults, although we cannot exclude that some of these fractures relate to the cooling of the diorite. The remaining fracture sets may be modern, and have formed either by stress relief during denudation^20^, or through Fe oxidation.

**Figure 8:**
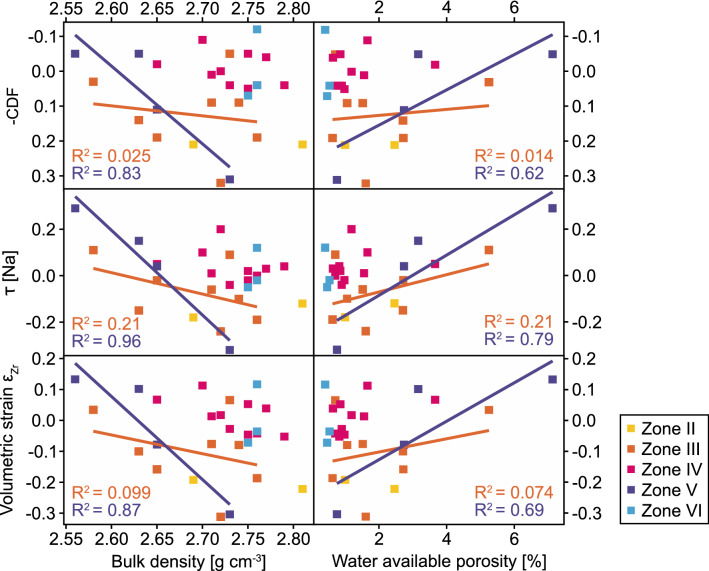
Chemical weathering indicators CDF and τ, and volumetric strain ε_Zr_ versus bulk density and water-available porosity. Chemical loss in the more strongly weathered zones III and V (orange and purple) shows a significant dependance on density and porosity, whereas less weathered (pink) and unweathered (blue) zones IV and VI shows no dependance. Physical weathering indicated by volumetric strain ε_Zr_ shows a dependence on density and porosity but variations in porosity cannot completely traced back to an increase in strain. More coherent and therefore less weathered samples are over-represented because the petrophysical analysis could not be conducted on disintegrated samples.

The described events have preconditioned the distinct weathering zones (Figs. 5, 6). The unweathered zone IV is identical in all parameters to zone VI (bedrock). Zone III and V represent two weathered intervals, as indicated by mass loss (negative CDF), increased surface area, loss of soluble elements Na and Ca (τ), and volumetric dilation (positive strain ε) that is correlated with increasing porosity (Fig. 8). Porosity likely forms by dissolution of plagioclase and hornblende without precipitation of pore-filling secondary minerals. In these zones, Fe oxidation, for example in biotite, is the most likely explanation for the increase in volume. Positive volumetric strain is found in zone V (Fig. 5f). In contrast, zone III shows a collapse of the profile indicated by negative volumetric strain. As this is an unlikely process, we assume a bias in Zr concentration caused by a different bedrock type.

In zone II, the topmost 7 m, all elements get significantly depleted except for U (Figs. 6, 7a). Because these samples consist of incoherent saprolite, most weathering indicators exceed those in the deeper alteration zones III and V, and because similar mass losses have been found in soil profiles over the same rock type close to the drill site^29^, we attribute this increased weathering intensity to decreasing lithostatic pressure during exhumation^47^, root wedging, and infiltration of meteoric fluid rather than this zone merely reflects a repetition of the deep features^48^.

The first weathering reaction taking place in the rock matrix in the vicinity of the faults is then in situ Fe oxidation of Fe-bearing minerals like biotite and hornblende which induces an increase in volume^15^. Fe oxidation of these minerals produces strain which in turn can lead to the formation of weathering-induced micro-fractures^12,14^.

That in situ Fe oxidation of bulk rock represents the dominating alteration process throughout the core is supported by the citrate bicarbonate dithionite extractable Fe. This fraction represents the reducible Fe(III) (oxyhydr)oxides and reflects the general trend indicated by the Fe(III)/Fe_total_ ratios. The amount of reducible and poorly crystalline oxides is substantial: it represents about 10–20% of the Fe pool of the entire drilling core and is particularly prominent in the highly altered zone V. However, the total amount of extractable Fe is higher than reported in Oeser et al.^29^. This is likely a result of increased extraction efficiency from the smaller grain size of our powdered rock samples compared to their sieved soil samples. Yet this substantial oxidation cannot unequivocally be attributed to weathering as sole cause. Hydrothermal alteration, as evidenced by martitised magnetite in association with chlorite and laumontite, might have led to some of the pronounced Fe oxidation, and would have pre-conditioned the deep rock for later weathering.

Following from the sum of these observations we group the alteration features into three categories: (1) obviously hydrothermal, characterised by minerals clearly of hydrothermal origin like chlorite, laumontite, and sericite. (2) Obviously weathering-related, mainly characterised by chemical mass loss found in the upper 7 m towards the surface. (3) Not unequivocally attributable, as characterised by Fe oxides that can be of either hydrothermal or weathering-induced origin. The origin of these Fe oxides is subject of future studies.

### Implications

The absence of a continuous weathering gradient in the drill core implies that here water flow through porous media might not be the main driver of weathering. We rather hypothesise that advection of fluids and gases through tectonic fractures sets deep weathering at multiple weathering fronts, since we found elevated degrees of chemical depletion close to larger fractures. Furthermore, the high abundance of magnetite might serve as a redox couple for deep microbial communities, enhancing silicate weathering in the process. Compared to semi-arid climates, weathering zones in humid climate show a higher chemical depletion and a shallower weathering zone^12,15,16,26^. In such settings, the formation of secondary minerals might in fact reduce the porosity and thereby limits the depth of fluid infiltration to the reaction front^12,13^. In contrast, in Santa Gracia this is not the case due to the lack of secondary minerals.

Finally, at this stage we cannot fully discount that the deep weathering fronts at Santa Gracia are ancient features. For example, the development of deep weathering could possibly date back to before the aridification of the Atacama Desert initiated in the Miocene^49,50^. We regard this possibility as unlikely, however, such ancient weathering features may not have survived in the Chilean Coastal Cordillera due to the steady tectonic uplift, and likely ensuing erosion. For the drill sites’ steady state denudation rate of 29.6 t km^−2^ year^−1^, corresponding to about 11 m Myr^−1^, the entire weathering zone is turned over about every 7 Myr. Thus, the zone might contain only a brief memory of the Miocene. Yet, this slow turnover allows generation of the observed weathering features even with minute fluid flow.

## Methods

### Geophysical borehole logging

After coring, geophysical well logging was conducted including gyroscope, acoustic televiewer, spectral gamma ray, full wave sonic, induced polarization/ resistivity, vertical seismic profile (VSP), and single point resistance (SPR). Detailed data sets for all used tools are available in Weckmann et al.^42^. The televiewer data (acoustic Televiewer BHTV 42, Electromind S.A., Luxembourg) was spatially oriented using a north-seeking Fiber Optic Gyro (Beijing Liuhe Greatness Technology, China). Representing the dip directions of structures, the azimuth was measured clockwise with respect to magnetic North. Azimuth and dip information allows to investigate fractures, borehole breakouts, and information on lithological features and boundaries. It utilizes the surface reflectivity of the wall in a fluid-filled borehole and the reflectance of ultrasonic pulses. The deviation from verticality between the surface drill location and the total depth of 87.2 m was about 1.3 m Southeast.

### Sample preparation and processing

Directly following the retrieval of each 1.5 m core run from the well, 20–30 cm long samples were separated from the core runs by using an angle grinder, hammer and chisel under sterile conditions. Samples were immediately stored in cooled (4 °C) vacuum-sealed bags. After shipment, we divided the samples by using a mechanical rock trimmer into a sample used for geochemical analyses (3–4 kg) and an archive sample that comprises the outer part of the core. The inner part of the core (0.5–1 kg), exposed to the lowest amount of drill fluid, is used as a sample for geomicrobiological analyses since a contamination by the drilling fluid should be minimal. Geochemical samples were further processed using a jaw breaker and subsequently a ball mill whereas microbiological samples were ground to a final grain size of < 2 mm by using a flame-sterilised disk mill. Every instrument in direct contact with the sample was first cleaned with ethanol and then treated with a Bunsen burner. The size of the samples and depth interval sampled (20–30 cm) are considered to average out variations, being representative in mineral abundance and fracture occurrence for the respective depth intervals.

### Contamination control

To assess the infiltration of drilling fluid into the drill core, a tracer was added to the fluid. We used a well-established protocol^40^, employing a fluorescent pigment dispersion (SPL-594 N, Day-Glo, Cleveland OH, USA) and detection by fluorescence microscopy. Ranging from 0.25 to 0.45 µm, the pigment particles are similar sized to environmental microorganisms. The pigment dispersion had a particle concentration of about 1 × 10^15^ particles L^−1^ in undiluted form and was added to the drilling fluid that obtained a final particle concentration between 1.1 × 10^11^ to 6 × 10^11^ particles L^−1^. Particle concentration in the drilling fluid was checked and adjusted regularly. Each time a core was retrieved from the well, a drilling fluid sample (“liner fluid”) was collected from the liner into a 15 mL centrifuge tube. A subsample of 0.5 mL was mixed with 9.5 mL particle-free water and 10 µL of the diluted sample were filtered on 0.2 µm polycarbonate membrane filters (Whatman Cyclopore) resulting in at least 200 countable particles using a Leica DM2000 fluorescence microscope with a UV filter set (Leica Filter Cube A, excitation BP340–380 nm, dichromatic mirror 400 nm, suppression LP425 nm). To estimate drilling fluid infiltration into inner core sections, about 250 mg of previously homogenised, crushed inner core samples were ground in a porcelain mortar and suspended in 1 mL MilliQ water. The suspension was shaken vigorously for 30 min and allowed to settle for 5 min. Tracer particles do not settle because of their small size and specific gravity of 1–1.1 g mL^−1^. 200 µL of the supernatant were then processed and analysed using the same method as for the liner fluid described above. Particle counts were converted to volume of drill fluid infiltration per mass of material, considering liner fluid concentration and a reduced count efficiency for this sample preparation, caused by mineral particles covering tracer particles and quantified by control experiments. The detection limit of the method is 0.02 µL drilling fluid infiltration per gram of sample.

### Element concentrations and modal mineral composition

Element concentrations were determined with inductively coupled plasma mass spectrometry (ICP-MS) and inductively coupled plasma optical emission spectrometry (ICP-OES) in a commercial laboratory (Activation Laboratories Ltd., Canada). On pulverised samples (< 74 μm), lithium metaborate and tetraborate fusions were performed using a robotic system. Sample analysis was performed by ELAN 6000, 6100 or 9000 ICP-MS (Perkin Elmer Sciex). For data quality control, replicates were analysed every 17 samples and reference materials were measured before and after every sample batch (reference: actlabs.com, 2020). The measured element concentrations were corrected for the loss on ignition (LOI):

1 $$\left[ X \right]_{corr} = { }\frac{{\left[ X \right]_{measured} *SUM_{measured} }}{{SUM_{{measured{ }}} - \left( {LOI_{measured} - { }\overline{LOI}_{bedrock} } \right)}}$$

where $\left[ X \right]_{corr}$ and $\left[ X \right]_{measured}$ are the corrected and the measured concentration of an element, respectively, $SUM_{{measured{ }}}$ is the sum of all analysed elements (without CO_2_ and LOI), $LOI_{measured}$ is the measured loss on ignition of a sample, and $\overline{LOI}_{bedrock}$ the averaged loss on ignition of bedrock samples.

The modal mineral composition of a bedrock sample was investigated using a micro X-ray fluorescence device (µ-XRF M4 Tornado, Bruker, USA) at Technische Universität Berlin. An area of 57 times 57 mm of a bedrock sample slab (cut perpendicular to the drilling direction) was mapped. A measuring spot size of 20 µm with a distance between spots of 50 µm were chosen and the integration time was 30 ms point^−1^. The maps were analysed with the open-source image processing program ImageJ and element combinations were attributed to the different minerals (i.e. calculation of area shares which are occupied by the respective element combinations). The modal mineral composition is complemented by ordinary point counting with an optical light microscope.

### Fe(III)/Fe_total_ ratios

To determine Fe(III)/Fe_total_ ratios, we used a colorimetric method as described by Schuessler et al.^51^. 8–10 mg of powdered sample aliquots were decomposed in a HF-vanadate mixture using V^5+^. After addition of 2:2′ bipyridyl solution, Fe(II) concentrations were measured on 10 mL of this solution by spectroscopy using a UV/VIS SPEKOL 1500 (Analytik Jena, Germany) in 1 cm transmission cells at 523 nm. After the Fe(II) measurement, hydroxylamine hydrochloride was added to the solution. Hydroxylamine hydrochloride acts as reducing agent and converts all Fe^3+^ to Fe^2+^ such that total Fe is obtained. By dividing the measured absorbances of Fe^2+^ and total Fe, the Fe(II)/Fe_total_ ratio and the Fe(III)/Fe_total_ ratio can be calculated. Weighing and dilution errors thus cancel out and uncertainties are primarily from spectroscopic measurements. Reference materials (GA and AC-E granite reference materials) and procedure blanks were run for quality control. Repeated measurements of standard solutions result in an uncertainty of ± 0.02 (2SD) for Fe(III)/Fe_total_ ratios.

### Oxide extractions

We extracted Fe in duplicates following a modified protocol of Mehra and Jackson^52^ using citrate bicarbonate dithionite (CBD; pH 7). CBD extracts crystalline and poorly crystalline iron (easily reducible oxides) such as ferrihydrite, goethite, and powdered hematite^48^. For each sample, 30 mL CBD were added to 0.5 g powdered samples from 1 to 86 m depth. Extractions were done on a rolling shaker at 10 rpm for 24 h anoxically in the dark. Extractions were centrifuged at 4000 rpm for 10 min, the supernatant removed anoxically and fixed with 1 M HCl in 1:10 dilutions. Finally, we quantified Fe_CBD_ -concentrations with the ferrozine assay and converted it into mg Fe g^−1^ rock powder. Al and Si concentrations were determined on the supernate by microwave plasma atomic emission spectrometry (MP-AES), and Fe concentrations by the ferrozine assay. All concentrations were converted into mg element g^−1^ rock powder.

### In situ ^10^Be

We processed samples for in situ ^10^Be separation in the *HELGES* laboratory at the GFZ German Research Centre for Geosciences (Potsdam, Germany) using the revised methods of von Blanckenburg et al.^53^. For ^10^Be analysis, we added ca. 150 μg of ^9^Be carrier to each sample. For this carrier, we determined a ^10^Be/^9^Be ratio of 3.4 × 10^–15^ (± 4.4 × 10^–15^) which we used for blank correction of measured ^10^Be/^9^Be ratios. After Fe and Be column chemistry and alkaline precipitation, Be was oxidised and pressed into accelerator mass spectrometer (AMS) cathodes and ^10^Be/^9^Be ratios were measured at the AMS at the University of Cologne relative to standards KN1-6-2 and KN1-5-3 (having nominal ^10^Be/^9^Be ratios of 5.35 × 10^–13^ and 6.32 × 10^–12^, respectively; which are consistent with the 07KNSTD standardization.

In order to derive denudation rates *D* (Eq. 2), we calculated nuclide production using the time-dependent scaling scheme of Lal/Stone (St)^54^ calibrated to a sea-level high latitude (SLHL) neutron spallation ^10^Be production rate of 4.01 at g^−1^ year^−1^ and solved the equation for *D*,

2 $$\left[ {{}_{{}}^{10} Be} \right] = \frac{{P_{N} }}{{\lambda + \frac{D}{{{\Lambda }_{N} }}}} + \frac{{P_{\mu } }}{{\lambda + \frac{D}{{{\Lambda }_{\mu } }}}}$$

where $[{}_{{}}^{10} Be]$ is the measured ^10^Be nuclide concentration (at g_(Quartz)_^−1^), $P_{N}$ is the scaled ^10^Be neutron production rate and $P_{\mu }$ that for muons (at g_qtz_^−1^ year^−1^)), ${\Lambda }_{N}$ and ${\Lambda }_{\mu }$ are the e-folding absorption lengths for neutrons and muons, respectively (g cm^−2^), and $\lambda$ is the decay constant of ^10^Be (5 × 10^–7^ year^−1^). The absorption of muons is calculated by a single exponential function derived from Beacon Heights, Antarctica^55^. The term $\rho /\Lambda$ is often replaced by z*, the absorption depth scale (cm), which is the distance over which the cosmic-ray flux decreases over the *e-*folding length, or 63%. This vertical distance, divided by the denudation rate, gives the integration timescale of the method.

### Density, porosity, water-available porosity

Buoyancy, He porosimetry, and water porosimetry were used to determine density, porosity, and water-available porosity of physically coherent samples. We first calculated a samples’ bulk Volume $V_{bulk }$ by assuming $V_{bulk } = V_{water}$, with $V_{water}$ being volume of the fluid displaced by a rock sample.

3 $$V_{bulk} = V_{water} = \frac{{m_{water} }}{{\rho_{{{\text{fluid}}}} }}$$

here $m_{water}$ is the mass of the displaced fluid (e.g. water) and $\rho_{{{\text{fluid}}}}$ is the density of the fluid at a certain temperature. With this volume $V_{bulk }$, the sample density $\rho_{bulk}$ can be calculated:

4 $$\rho_{bulk} = \frac{{m_{{{\text{dry}}}} }}{{V_{bulk} }}$$

where $m_{{{\text{dry}}}}$ is the dry rock sample mass. The estimated uncertainty of the density measurements is < 0.3%.

He pycnometry was used to estimate the matrix volume $V_{matrix}$. of the solid part of a sample. The measurements were conducted on the same samples as used for density determination. Samples were dried and weighed before placing them in an AccuPyc 1030 He pycnometer (Micromeritics, USA). The sample was placed into a sealed chamber which was then flooded with He. Because the volume of the chamber is known, $V_{matrix}$. can be calculated from the difference in chamber fill volume. Porosity $\phi_{He}$ can be obtained with:

5 $$\phi_{{{\text{He}}}} = \frac{{V_{{{\text{bulk}}}} - V_{{{\text{matrix}}}} }}{{V_{{{\text{bulk}}}} }}$$

Uncertainty of the measurements are < 0.03% of gas displacement reading plus 0.03% for the chamber volume.

The water-available porosity measured with water porosimetry $\phi_{{{\text{con}}}}$ can be calculated from the sample weight before and after saturation with deionised water for 48 h:

6 $$\phi_{{{\text{con}}}} = \frac{{V_{{{\text{sat}}}} }}{{V_{{{\text{bulk}}}} }}$$

where $V_{{{\text{sat}}}}$ is the volume of pores saturated with water. The uncertainty of the connected porosity is < 0.14%.

For comparison we used the measured P wave velocity from the sonic log to derive a calculated density log. We applied a parameterised velocity-density relationship based on global petrophysical studies for sedimentary, igneous, and metamorphic rocks^56,57^.

### Specific surface area (SSA)

The specific surface area (SSA) was determined by nitrogen gas sorption using the BET equation^58^. About 3 g of powdered sample material was degassed in a Vac Prep Q61 Sample Degas System (Micromeritics, USA). During the degassing process, bedrock samples were heated up to 250 °C, samples from zones C-E up to 60 °C for durations of 8–20 h, and samples from zone A and B up to 120 °C for 16 h. After degassing, the SSA was measured by N_2_ sorption in a Gemini VII Surface Area and Porosity analyser (Micromeritics, USA). The uncertainty of the SSA measurement is ~ 1.1% based on repeat analysis (*n* = 3, relative SD = 0.29%) of a certified reference material (Carbon Black, SSA_BET_ = 21.52 ± 0.75 m^2^ g^−1^). The detection limit is 0.01 m^2^ g^−1^.

### Calculations of weathering indicators

Weathering indicators have been calculated from elemental concentrations. The chemical depletion fraction (CDF) represents the relative mass loss due to chemical weathering of the bedrock^59^:

7 $$CDF = 1 - \frac{{[X_{i} ]_{{\text{p}}} }}{{[X_{i} ]_{{\text{w}}} }}$$

where $[X_{i} ]_{{\text{p}}}$ and $[X_{i} ]_{{\text{w}}}$ are the concentration of the immobile element [$X_{i} ]$ in the weathered saprolite (${\text{w}}$) and the unweathered parent bedrock (${\text{p}}$), respectively. We used Zr as immobile element. Zero elemental loss during chemical weathering from parent bedrock to saprolite yields a CDF of zero. We used four samples without visible weathering features as the average value for bedrock. In granitic rocks, the CDF can attain a maximum value of 0.5 if weathering is complete and only quartz, clay minerals, and secondary oxides remain. The loss of individual elements during chemical weathering can be calculated with the mass transfer coefficient $\tau_{j, w}$ (tau)^60^. The ratio of the concentration of $X$ in weathered rock to that in parent bedrock material depends on the loss of the element $j$, but also on the loss of other elements. Thus, the calculation $\tau_{{j, {\text{w}}}}$ includes concentrations of an immobile element $X_{{i,{\text{p}}/{\text{w}}}}$:

8 $$\tau_{{j, {\text{w}}}} = \frac{{[X_{j} ]_{{\text{w}}} [X_{i} ]_{{\text{p}}} }}{{[X_{j} ]_{{\text{p}}} [X_{i} ]_{{\text{w}}} }} - 1$$

where $[X_{j} ]_{{\text{w}}}$ and $[X_{j} ]_{{\text{p}}}$ are the concentrations of an element $j$ in weathered saprolite and unweathered parent bedrock. $\tau = 0$ indicates zero loss of an element relative to the bedrock, $\tau < 0$ shows loss of a certain element whereas $\tau > 0$ means gain. Because the sense of $\tau$ is opposite to that of CDF we plot—CDF in Figs. 5 to facilitate comparison.

A weathering rate $W$ assuming steady state denudation is calculated from the cosmogenic nuclide-derived soil denudation rate $D$ and $CDF$:

9 $$W = D*CDF$$

### Volumetric strain ε_Zr_

To calculate the volumetric strain ε_Zr_, we used the average bedrock Zr concentration $[X_{i} ]_{{\text{p}}}$ and the average bedrock bulk density $\rho_{{bulk_{p} }}$ as well as the Zr concentration $[X_{i} ]_{{\text{w}}}$ and the bulk density $\rho_{{bulk_{w} }}$ of a weathered sample (Tables S1, S2)^43^:

10 $$\varepsilon_{Zr} = \frac{{\rho_{{bulk_{{\text{p}}} }} }}{{\rho_{{bulk_{{\text{w}}} }} }}*\frac{{[X_{i} ]_{{\text{p}}} }}{{[X_{i} ]_{{\text{w}}} }} - 1$$

Values close to zero indicate isovolumetric weathering. Positive values show dilation whereas negative values indicate a collapse of a profile.

## Data Availability

The datasets generated and analysed during the current study are available in the GFZ Data Services repository, 10.5880/GFZ.3.3.2021.002.
